# How to Make Plastic Surfaces Simultaneously Hydrophilic/Oleophobic?

**DOI:** 10.1021/acsami.3c06787

**Published:** 2023-06-16

**Authors:** Yihan Song, Michaela Dunleavy, Lei Li

**Affiliations:** Department of Chemical & Petroleum Engineering, University of Pittsburgh, Pittsburgh, Pennsylvania 15261, United States

**Keywords:** hydrophilic/oleophobic, PMMA, polystyrene, polycarbonate, UV/Ozone, perfluoropolyether, anti-aging

## Abstract

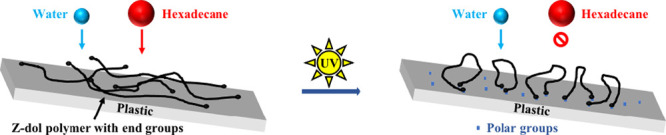

Hydrophilic/oleophobic surfaces are desirable in many applications including self-cleaning, antifogging, oil–water separation, etc. However, making plastic surfaces hydrophilic/oleophobic is challenging due to the intrinsic hydrophobicity/oleophilicity of plastics. Here, we report a simple and effective method of making plastics hydrophilic/oleophobic. Plastics, including poly (methyl methacrylate) (PMMA), polystyrene (PS), and polycarbonate (PC), have been coated with a perfluoropolyether (PFPE) (i.e., commercially known as Zdol) via dip coating and then irradiated with UV/Ozone. The contact angle measurements indicate that the treated plastics have a lower water contact angle (WCA) and higher hexadecane contact angle (HCA), i.e., they are simultaneously hydrophilic/oleophobic. The Fourier transform infrared (FTIR) results suggest that UV/Ozone treatment introduces oxygen-containing polar groups on the plastic surfaces, which renders the plastic surfaces hydrophilic. Meanwhile, more orderly packed PFPE Zdol molecules, which is due to the UV-induced bonding between PFPE Zdol and the plastic surface, result in the oleophobicity. Moreover, the simultaneous hydrophilicity/oleophobicity of functionalized plastics does not degrade in aging tests, and they have superior antifogging performance and detergent-free cleaning capability. This simple method developed here potentially can be applied to other plastics and has important implications in the functionalization of plastic surfaces.

## 1 Introduction

Surfaces more wettable to water than to oil, also known as simultaneously hydrophilic/oleophobic surfaces, are desirable in many applications such as anti-fogging,^1−6^ oil–water separation,^3,6−13^ antifouling,^6,14,15^ self-cleaning,^1,2,6,13,16,17^ etc. However, fabricating such surfaces is challenging because of the higher surface tension of water than oil, leading to the fact that the water contact angle (WCA) is greater than the oil contact angle on most solid surfaces.^18^ In recent years, one strategy to achieve such special wettability is to chemically modify the surface with coatings containing both hydrophilic and fluorinated (i.e., oleophobic) segments, which has been demonstrated by several groups.^1−3,7,10,11,14,15,19−40^ Previously, we have dip-coated hydrophilic substrates, i.e., silicon wafer and glass, with a nanometer-thick perfluoropolyether (PFPE) with hydroxyl end groups.^4,41^ It was found that the interaction between PFPE’s hydroxyl end groups and substrates’ polar groups induces a more orderly packed polymer conformation, where the appropriate “hole” size between polymer chains allows water molecules, which has a smaller molecular size, to penetrate through and “see” the polar groups on substrates. However, hexadecane molecules cannot penetrate through the same “holes” due to their larger molecular size and thus “see” the fluorinated segments on the top.^4,41^ Consequently, WCA on such PFPE-coated silicon wafer/glass is lower than the hexadecane contact angle (HCA). Although simultaneous hydrophilicity/oleophobicity can be achieved on hydrophilic substrates, it has not been reported on plastic substrates with intrinsic hydrophobicity/oleophilicity, to the best of our knowledge.

Plastics are one of the most critical materials due to their corrosion resistance, lightness, low cost, and excellent mechanical and optical properties.^42−45^ It was predicted that the consumption of engineering thermoplastics will reach up to 29.1 million metric tons by 2020.^42^ Particularly, the roles a plastic material plays in wastewater treatment, antifogging surface, and microfluidic devices have been increasingly stressed. Some examples are polyvinylidene fluoride (PVDF) for oil–water separation membranes,^46^ poly (methyl methacrylate) (PMMA) or polycarbonate (PC) for anti-fogging goggles and windshield,^47,48^ and polydimethylsiloxane (PDMS) or polystyrene (PS) for microscale cell-based systems.^49^ However, the inherent hydrophobicity/oleophilicity of plastics requires surface modification when it comes to these applications where hydrophilicity/oleophobicity is desirable.^44,45,50^ Our previous studies^4,41^ indicate that the polar substrate, i.e., hydrophilic ones, is the key condition for PFPE coating to be more wettable to water than to oil. Since most plastics are hydrophobic in nature, it is challenging to make plastic simultaneously hydrophilic/oleophobic.

The approaches to modify plastic’s surface wettability can be divided into two categories: surface activation and surface coating.^50^ Oxygen plasma and UV/Ozone are two of the most common surface activation treatments to enhance the surface energy of plastic by introducing oxygen-containing polar groups.^44,48,51,52^ However, it has been shown by many previous reports that such enhancement will degrade with time, and the WCA of aged polymer surfaces will increase toward the original value, also known as hydrophobic recovery.^44,48,51,52^ Surface coating can be achieved via either vapor deposition or wet chemical coating process.^50,53^ Chang et al. spin-coated PMMA with a UV-curable bilayer structure composed of a silica-embedded crosslinked network of dipentaethritol hexaacrylate (DRHA) as the hydrophobic barrier, and a hydrophilic top layer synthesized from Tween-20, isophorone diisocyanate (IPDA), and 2-hydroxyethyl methacrylate (2-HEMA).^54^ They reported a WCA of ∼0° and superb antifogging performance as increasing the Tween-20 content at the expense of mechanical strength and coating adhesion.^54^ Zilio et al. demonstrated the hydrophilization of thermoplastics including PDMS and PC with two different coatings of poly(dimethylacrylamide) copolymerized with either N-acryloyloxysuccinimide (NAS) or glycidyl methacrylate (GMA).^53^ Not only was the WCA of all coated plastics decreased to ∼40°, these two coatings can effectively reduce the nonspecific adsorption of human serum albumin and bovine serum albumin.^53^ Although the effect of these methods will not diminish with time, their application is limited by the complicated coating chemistry and multistep procedures. More importantly, there has not been any report on functionalizing plastic substrates with enhanced oleophobicity, not to mention simultaneous hydrophilicity/oleophobicity.

In this paper, we report a simple and effective strategy to make plastics, including PS, PC, and PMMA, simultaneously hydrophilic/oleophobic. The plastics are first dip-coated with a nanometer-thick perfluoropolyether (PFPE) (i.e., Zdol) and then undergo UV/Ozone treatment. We showed here that, with the assistance of UV/Ozone treatment, Zdol-coated plastics become simultaneously hydrophilic/oleophobic. The results of contact angle measurement show that such functionalized plastics have a lower WCA and higher HCA, which remain almost unchanged after an aging period of up to 4 weeks. Their excellent anti-fogging performance and self-cleaning ability have also been demonstrated.

## 2 Results and Discussion

### 2.1 Simultaneous Hydrophilicity/Oleophobicity

#### 2.1.1 Contact Angle

**Figure 1:**
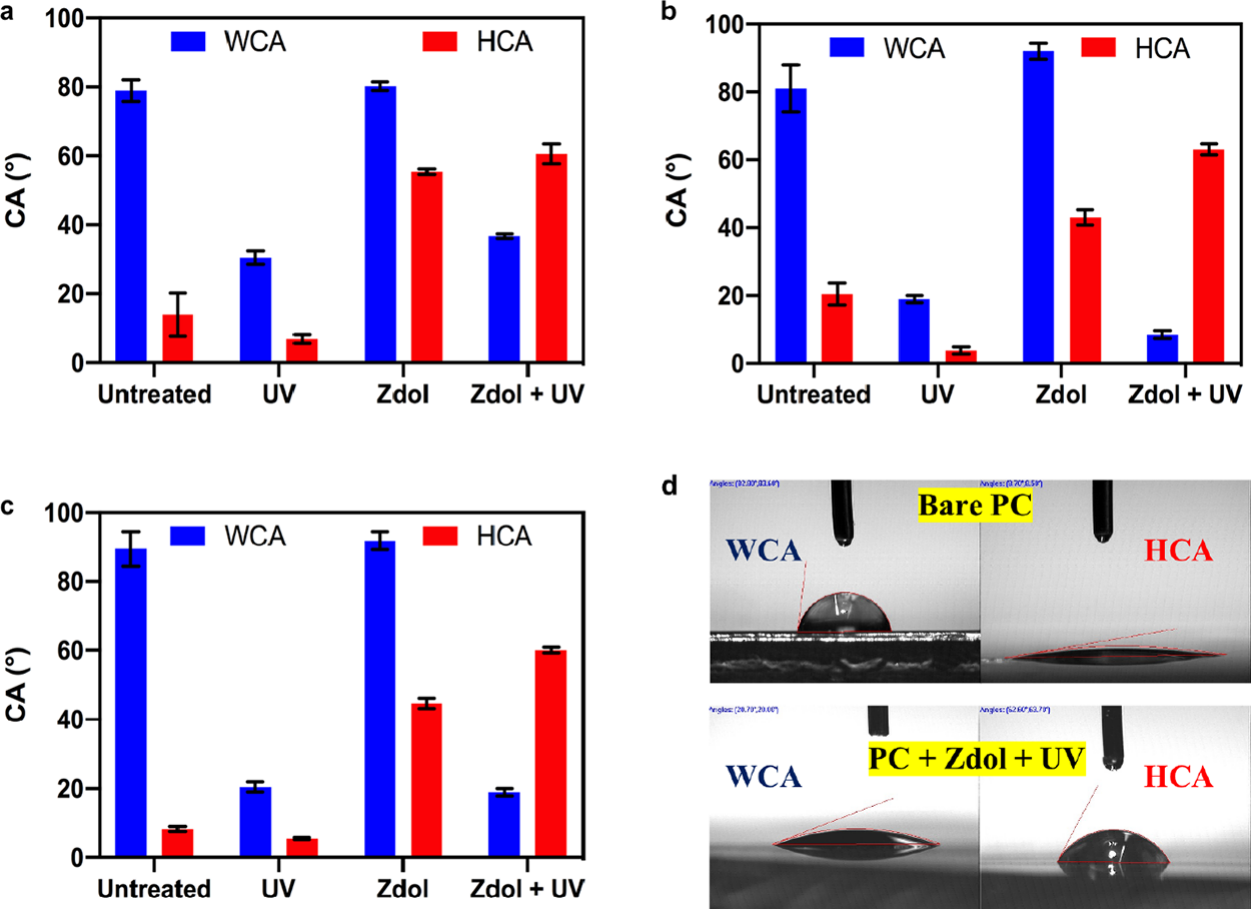
Contact angle of PMMA (a), PS (b), and PC (c) with different treatments, and the images of WCA and HCA on bare and functionalized PC (d).

The WCA and HCA of plastics with different treatments are shown in Figure 1. Bare substrates of PMMA, PS, and PC all have a higher WCA (>75°) and a lower HCA (<20°), indicating these plastics are intrinsically hydrophobic/oleophilic. After UV/Ozone exposure for 20 min, the WCA of 3 plastics decreases significantly to <30°, showing the hydrophilicity of plastic substrates has been effectively improved. As a simple oxidizing method, UV/Ozone treatment is good at removing contaminants from the surface of different materials.^55^ In UV/Ozone treatment, the 185 nm UV light produces ozone given the presence of oxygen, meanwhile 254 nm UV light excites hydrocarbon contaminants to generate free radicals which will react with ozone, producing volatile byproducts such as CO_2_ and H_2_O.^55^ At the same time, the treated surfaces are oxidized to create more oxygen-containing species which increase the surface energy and hydrophilicity.^44^ However, these UV-treated plastics are still oleophilic with an HCA of <10°. To increase their oleophobicity, 3 plastic substrates are dip-coated with PFPE Zdol (No UV/Ozone treatment), which increases their HCA to above 40° and also slightly increases their WCA (>80°). The addition of fluorinated coating, lowering the surface energy of plastics, is responsible for the increased HCA and WCA. The coating thickness measured is ∼1 nm (see Figure S5 in SI). Although Zdol coating enhances the HCA of 3 plastics by fluorinated polymer chain, their WCA remains higher than the HCA. Interestingly, after Zdol-coated plastics are treated with UV/Ozone for 20 min, their WCA drops to ∼35°, ∼20°, and ∼10° for PMMA, PS, and PC, respectively, which are almost equivalent to the WCA of UV-treated bare plastics. In the meantime, their HCAs increase to >60°, indicating plastic surfaces are rendered simultaneously hydrophilic/oleophobic successfully. Figure 1d exhibits the significant change in plastics’ surface wettability by representative images of WCA and HCA of PC before and after functionalization.

#### 2.1.2 ATR-FTIR

**Figure 2:**
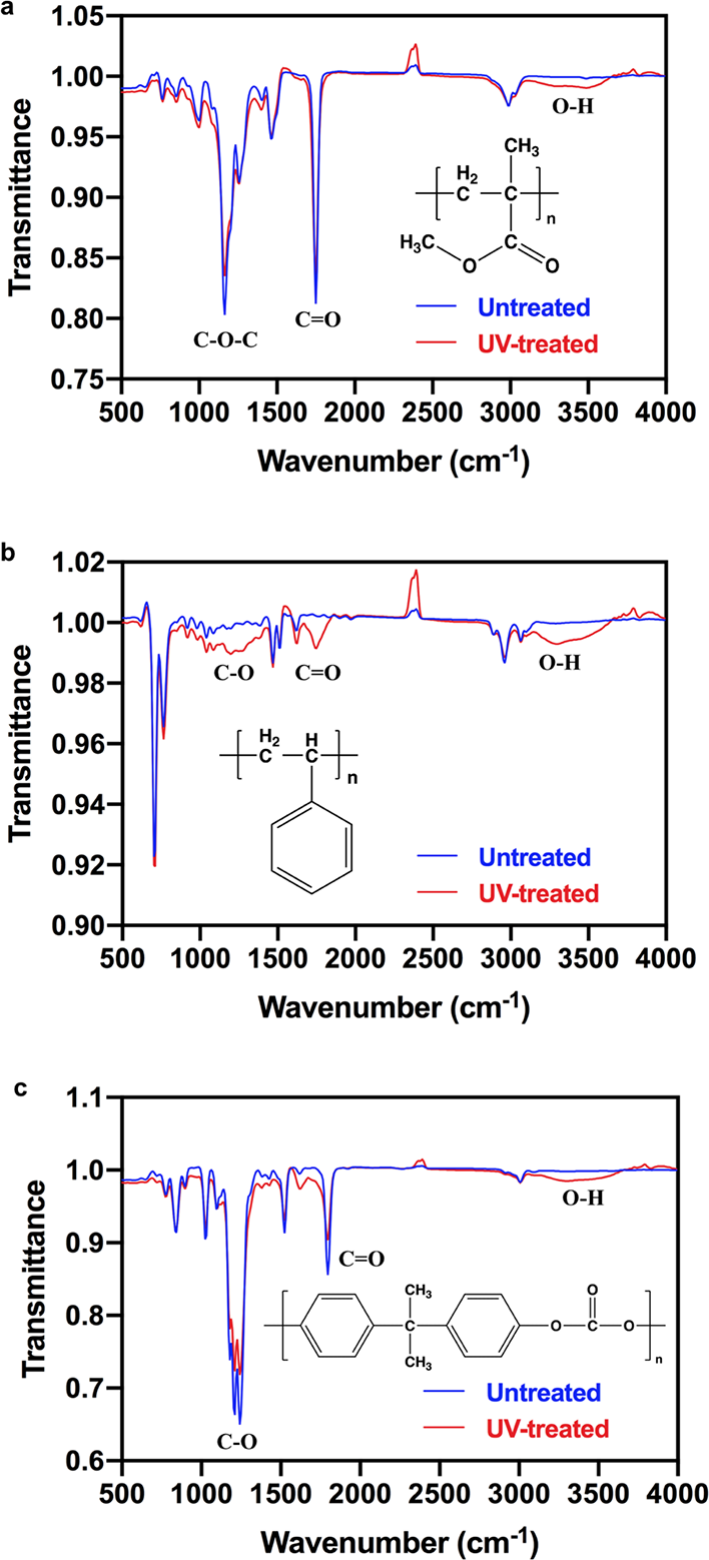
FTIR spectra of PMMA (a), PS (b), and PC (c) before and after UV/Ozone treatment. Insets are the chemical structures of 3 plastics.

In order to understand the effect of UV/Ozone treatment, the Fourier transform infrared (FTIR) spectra of untreated and UV-treated plastics were collected and are exhibited in Figure 2. In Figure 2a, the peaks located at 1757 and 1249 cm^–1^ are assigned to C=O and C–O–C stretching vibrations of ester groups of PMMA, respectively.^43,52^ After UV/Ozone treatment, the intensity of these two peaks noticeably decreases; meanwhile, higher intensities are observed at the peak shoulders in the ranges of 1000–1125 and 1600–1800 cm^–1^, suggesting the photolysis of ester groups yields oxygen-containing polar groups such as carboxylic acids, aldehydes, ketones, and alcohols.^43^ The appearance of the peak at 3000–3600 cm^–1^, which is attributed to OH stretching, also indicates the generation of hydroxyl groups after UV/Ozone treatment. Such chemical changes on PMMA surfaces have been shown to indicate the photodegradation and photooxidation of PMMA in previous studies. In brief, PMMA undergoes main-chain scission and side-chain scission under UV irradiation with a UV wavelength shorter than 320 nm, and the scission of ester side chains is more efficient and can also result in main-chain scission.^56−58^ The bond cleavages of the ester side group induced by photoexcitation produce radicals that can be oxidized in the presence of oxygen, leading to the β-scission.^56^ In the meantime, the formation of end products including ketones, aldehydes, carboxylic acid, and alcohols increases the surface hydrophilicity.^43^

The spectra of PS are shown in Figure 2b. Two characteristic peaks at 1468 and 765 cm^–1^ are due to deformational vibrations of a benzene ring and substituted benzene derivative, respectively.^45,59^ After UV/Ozone treatment, there are absorption bands showing up at 3000–3600 cm^–1^ (O–H), 1600–1800 cm^–1^ (C=O), and 1100–1300 cm^–1^ (C–O), which correspond to the oxygen-containing polar groups of photolysis products of PS. The photodegradation of PS is initiated by the excitation of benzene rings which can absorb light quanta and are transformed to singlet and triplet states, and the energy gained can be transferred to C–H and C–C bonds, leading to hydrogen abstraction and backbone scission.^60^ When oxygen is present, singlet oxygen can be formed by the energy transfer from the excitation of benzene rings to the ground state of molecular oxygen, and it will oxidize the polymer backbone and benzene rings simultaneously.^60,61^ The end products include acetophenone, phenol, mucondialdehyde, and keto-lactone type structures,^60,61^ which have oxygen-containing polar groups and thus increase the hydrophilicity of PS surface.

The spectra of PC in Figure 2c present damped characteristic peaks at 1796 cm^–1^ (C=O), 1523 cm^–1^ (C–C), 1242 cm^–1^ (C–O), and the growth of absorption band at 3000–3600 cm^–1^ (O–H) and 1600–1800 cm^–1^ (C=O) after UV/Ozone treatment. As for PC under UV irradiation, photo-Fries rearrangement is the primary process in which the scission of CO–O bonds leads to the rearrangement of polymer chains and the formation of phenylsalicylate and dihydroxybenzophenone units.^62−64^ Competitively, radicals generated by CO–O bond scissions may be oxidized in the presence of oxygen, which can initiate the photooxidation of PC by the abstraction of hydrogen from methyl groups.^63^ Then, the dimethyl sidechain photooxidation is induced by the photolysis of hydroperoxides formed there, resulting in the β-scission.^63^ The formation of end products including phenol, benzoic acid, acetic acid, formic acid, etc.,^63^ is confirmed by the FTIR spectra and causes the increased hydrophilicity mentioned earlier.

Therefore, the FTIR spectra of all 3 plastics after UV/Ozone treatment suggest an effective surface modification with the introduction of oxygen-containing polar groups which increase the plastics’ surface energy and hydrophilicity. The same conclusion can be drawn based on the FTIR spectra of Zdol-coated plastics before and after UV/Ozone treatment (see Figure S4 in SI)

#### 2.1.3 Proposed Mechanism

**Figure 3:**
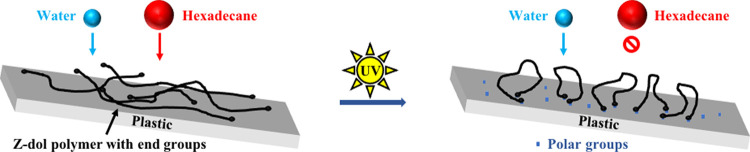
Schematic of Zdol polymers bonding to plastic substrates after UV/Ozone treatment.

Here we propose the mechanism of simultaneous hydrophilicity/oleophobicity induced by UV/Ozone treatment on Zdol-coated plastics. Based on our previous studies,^4,16,41^ the WCA and HCA of PFPE-coated solid surfaces highly depend on the intermolecular “hole” size of PFPE, which is largely determined by the PFPE-substrate interaction. When amorphous Zdol polymers are disorderly deposited onto plastic surfaces, as shown in Figure 3, the interchain distance is relatively large, thus leading to openings for hexadecane molecules to penetrate through the polymer film and “see” the plastic substrate, i.e., smaller HCA (∼40°) is observed. Once such surface is exposed to UV/Ozone irradiation, Zdol’s hydroxyl end groups tend to H-bond with the oxygen-containing polar groups (e.g., hydroxyl groups) created by UV/Ozone on plastic surfaces,^65,66^ resulting in more ordered packing of Zdol chains, as illustrated in Figure 3. Consequently, the interchain distance decreases, inducing higher resistance to the penetration of hexadecane and thus larger HCA (>60°). Previously, we have shown that, on hydrophilic substrates (e.g., silica), Zdol forms a much more ordered structure, which results in simultaneous hydrophilicity/oleophobicity.^4,41^ Here, UV/Ozone irradiation is shown to create polar groups on the plastic surfaces, which enable the simultaneous hydrophilicity/oleophobicity. In addition to H-bonding, the UV-initiated bonding between Zdol and plastic surfaces could also be covalent in nature as reported before.^16^ This could occur via two mechanisms. First, photoelectrons excited by UV illumination are emitted from the plastic substrate and cause the dissociation of Zdol molecules.^67^ Second, the photodissociation of Zdol molecules is directly induced by UV light.^68^

### 2.2 Aging Tests

**Figure 4:**
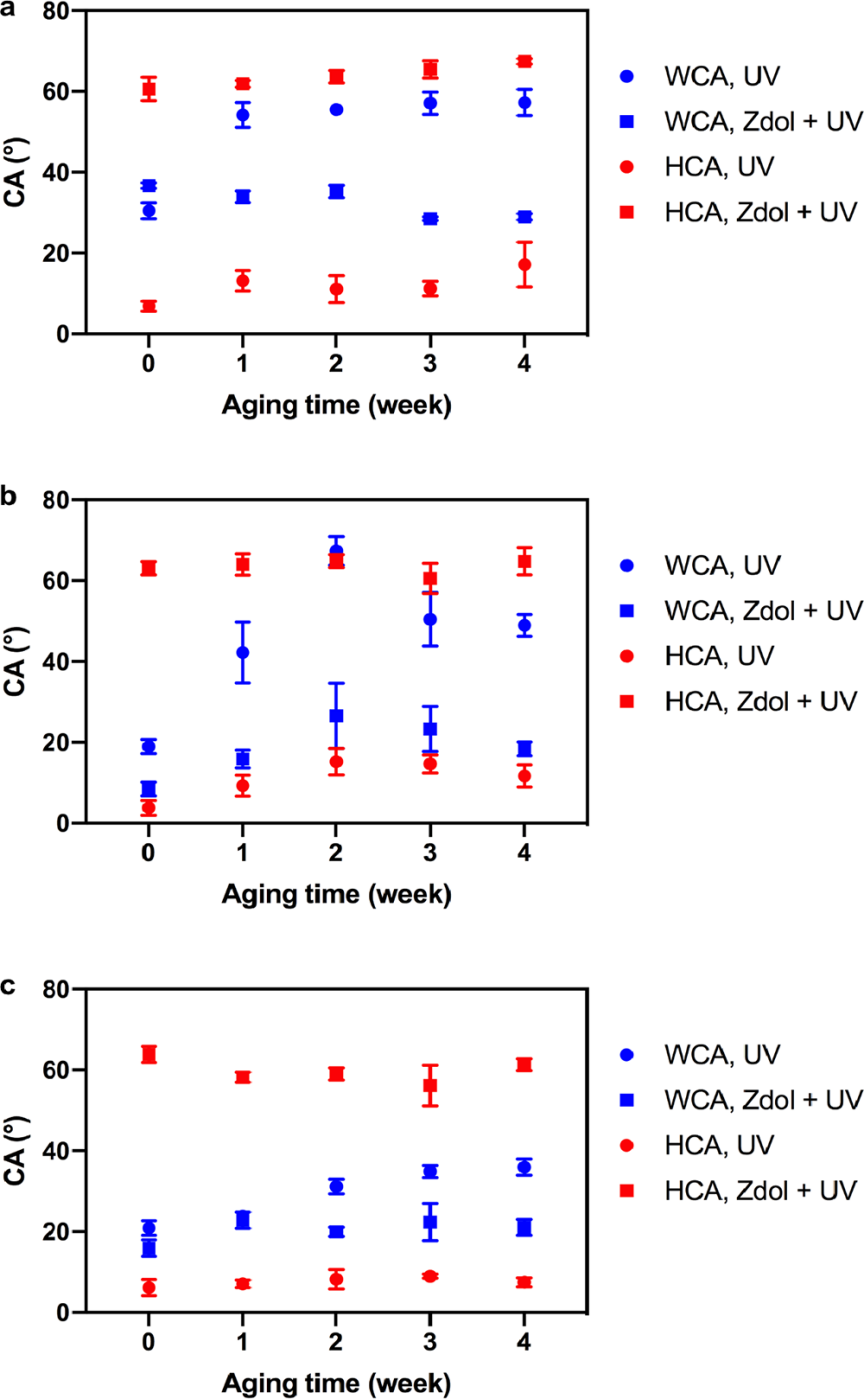
Contact angle of PMMA (a), PS (b), and PC (c) during the 4-week aging period after treatments.

It has been widely reported that plastics conventionally treated by plasma and UV/Ozone will experience hydrophobic recovery with time, i.e., their WCA increases back to the value before the treatment.^44,48,51,52^ It has been proposed that airborne hydrocarbon can adsorb on plastic surface and thus lowers the surface energy and increases the WCA.^55^ Aging tests were conducted on our Zdol-coated and UV/Ozone-treated plastics with simultaneous hydrophilicity/oleophobicity. The plastics with UV/Ozone treatment (No Zdol coating) were also tested as a control. As shown in Figure 4, the WCA of UV-treated plastics (No Zdol coating) increases by ∼20° for PMMA, ∼30° for PS, and ∼10° for PC after 4 weeks, which is consistent with previous reports. However, for Zdol-coated and UV/Ozone-treated plastics, there is little change in WCA after 4 weeks. This can be explained by the fact that the presence of PFPE Zdol coating slows down the airborne hydrocarbon contamination because of its oleophobicity.^4^ Meanwhile, there is no significant change in the HCA of Zdol-coated and UV/Ozone-treated plastics during 4 weeks as well, indicating the durable oleophobicity. Overall, the hydrophobic recovery of plastics can be effectively suppressed using our strategy of Zdol coating followed by UV/Ozone treatment.

### 2.3 Applications

#### 2.3.1 Antifogging

**Figure 5:**
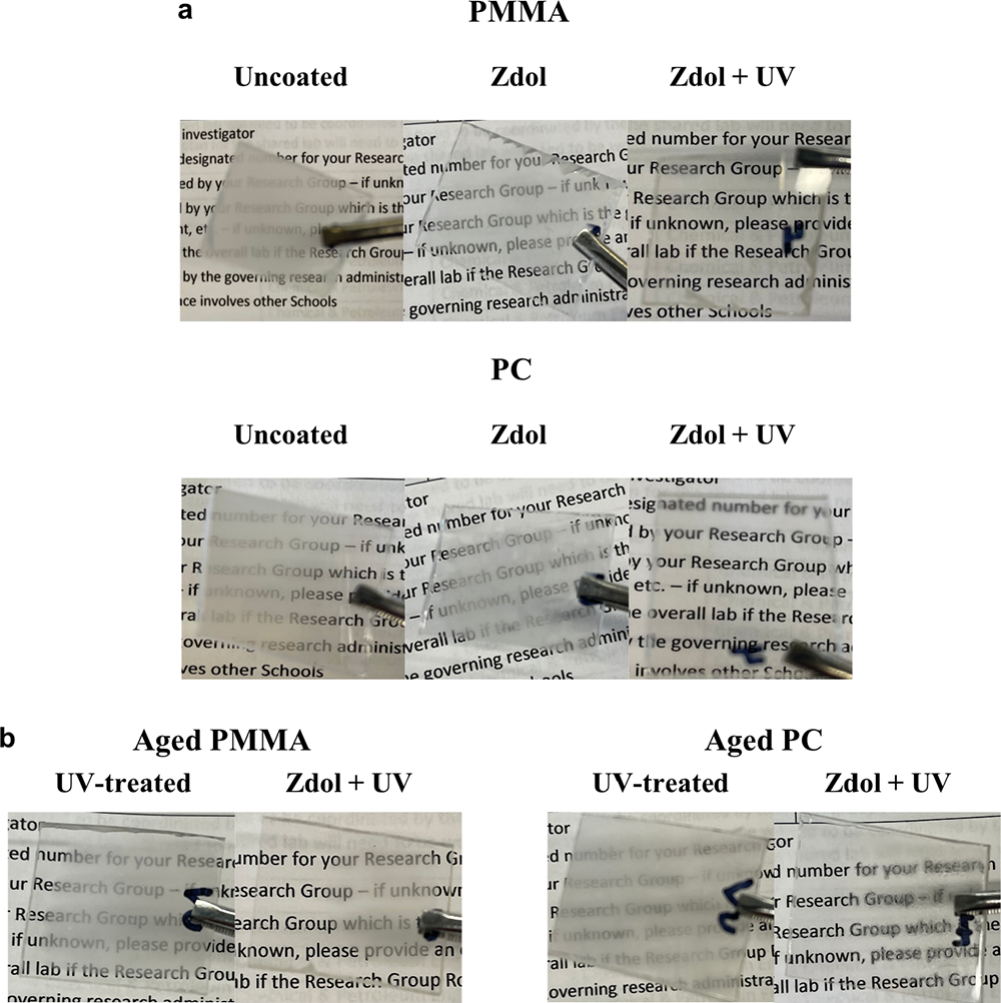
Antifogging performance of bare and Zdol-coated PMMA/PC with and without UV/Ozone treatment (a) and UV-treated and functionalized PMMA/PC after 4-week aging (b).

Antifogging surfaces are critical to camera lenses, goggles, and automobile windshields where the application of plastics, such as PMMA and PC, becomes more and more popular.^47^ Fogging resulted from water droplets beading up on those surfaces reduces the light transmission and thus degrades the performance.^4,16^ It has been found that, in order to prevent fogging, WCA on the surface should be lower than ∼40° so that a uniform water film instead of water beads is formed.^1,4^ Here, we tested the antifogging performance of our functionalized plastics where low WCA values have been demonstrated. Figure 5a shows that functionalized PMMA and PC have superior antifogging performance over bare and Zdol-coated plastics, in which the transparency of functionalized plastics after exposure to water steam is barely degraded due to their improved hydrophilicity. On the other hand, the aging effect on antifogging performance was tested on the UV-treated and functionalized PMMA/PC which had been stored in the ambient environment for 4 weeks after fabrication. As shown in Figure 5b, the aged PMMA and PC with full functionalization have a small amount of fog, while those only UV/Ozone-treated show severe fogging, which significantly reduces the transparency. The results can be explained by the fact that simultaneous hydrophilicity/oleophobicity can slow down the hydrophobic recovery of functionalized plastics by inhibiting airborne hydrocarbon contamination.^4,16^ Fogging occurs on UV/Ozone-treated plastics (No Zdol coating) after aging because their oleophilicity allows airborne hydrocarbon to adsorb onto the surfaces, which renders the surface increasingly hydrophobic and prone to be fogged with time. In contrast, our functionalized plastics remain hydrophilic in aging because the oleophobicity can reduce the hydrocarbon contamination, demonstrated above by a lower WCA compared to aged plastics with UV/Ozone treatment only, which does mitigate fog formation. Hence, this functionalization approach established in the current research is promising in antifogging application of plastics.

#### 2.3.2 Detergent-Free Cleaning

**Figure 6:**
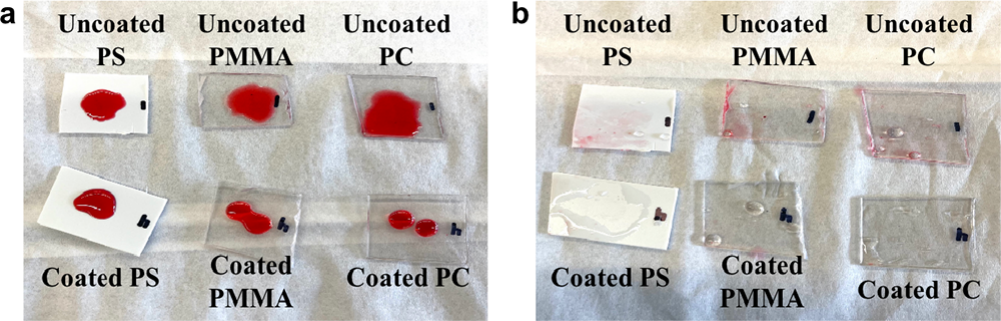
Detergent-free cleaning testing results of untreated and functionalized plastics: hexadecane droplets on plastic surfaces (a) and after rinsing with DI water (b).

Because simultaneously hydrophilic/oleophobic surfaces are more wettable to water than to oil, another potential application of such surfaces is in detergent-free cleaning, i.e., such surfaces contaminated by oil can be cleaned with just water.^1,2,16^ Thus, the use of detergent is no longer needed, which reduces the detergent-related pollution in the environment and the consumption of petroleum that is the raw material of detergent.^16^Figure 6 presents the results of self-cleaning testing of our functionalized plastics. The hexadecane droplets dyed red on Zdol-coated plastics with UV/Ozone treatment can be easily removed by water rinsing, while hexadecane residual remained on bare plastic surfaces after washing. This demonstrates the excellent self-cleaning capability of functionalized plastics due to their simultaneous hydrophilicity/oleophobicity.

## 3 Conclusions

A simple approach, including dip-coating of a nanometer-thick PFPE Zdol followed by UV/Ozone treatment, has been demonstrated to be effective in making intrinsically hydrophobic/oleophilic plastic surfaces simultaneously hydrophilic/oleophobic. FTIR results show that the UV/Ozone treatment generates oxygen-containing polar groups on plastic surfaces and thus significantly improves the hydrophilicity. Meanwhile, UV/Ozone treatment induces the H-bonding between the PFPE Zdol and the plastic substrates, which results in more ordered packing of PFPE chains. Consequently, the interchain distance between Zdol polymers decreases so that large oil molecules cannot penetrate the coating and only “see” the fluorinated segments staying on top, which enhances the oleophobicity. It has also been demonstrated that the simultaneous hydrophilicity/oleophobicity does not degrade in the aging test, which addresses the issue that hydrophobic recovery always occurs in the long run to plastics treated by UV/Ozone only. Moreover, we have demonstrated that the functionalized plastics have excellent antifogging performance and detergent-free cleaning capability. The finding here establishes a simple and effective approach to make plastics simultaneously hydrophilic/oleophobic, which has important implications in many applications involving plastics.

## 4 Experimental Section

### 4.1 Materials and Sample Fabrication

The PFPE polymer, Zdol, was obtained from Solvay Solexis Inc. 2,3-Dihydrodecafluoropentane, commercially known as Vertrel XF, was purchased from Miller Stephenson Chemical Co., and it was used as the solvent for preparing Zdol solutions. Hexadecane, acetone, and Oil red dye were purchased from Sigma Aldrich. Isopropanol (IPA) was obtained from Fisher Scientific. All the chemicals were used as received. Deionized (DI) water was produced from a Millipore Academic A10 system (total organic carbon lower than 40 ppb). Plastic substrates, including PMMA, PS, and PC were purchased from McMaster-Carr, and cut into 2 cm × 3 cm pieces. 1 g/L Zdol solution was prepared by mixing 1 g neat Zdol and 1 L Vertrel XF, with which Zdol was coated on 3 plastics by a dip-coating procedure reported in previous studies.^4,16,41^

### 4.2 UV/Ozone Treatment

UV/Ozone treatment was performed with a BioForce Nanosciences UV/Ozone Procleaner which emits a high-intensity UV light (110VCA, 50/60 HZ, 0.5A, and 1 PH) with 185 and 254 nm wavelengths. The treatment in this study was conducted under room temperature (∼24 °C) in ambient air for 20 min.

### 4.3 Contact Angle Measurements

The WCA and HCA on plastic substrates were measured using a VCA optima XE (AST Production Inc.) system in an ambient environment. In testing, an image of a liquid–solid interface was photographed after a sessile liquid droplet of 1 μL was deposited on substrates, and the value of the contact angle was determined automatically by the VCA software. All the reported contact angles were determined by averaging at least 3 measurements at different locations of the tested sample.

### 4.4 ATR-FTIR

The FTIR spectra of plastic substrates were obtained by employing a Bruker Vertex-70LS FTIR in the attenuated total reflection mode with Ge 20× ATR objectives. The spectra were collected for 64 scans at 16 cm^–1^ resolution in the range between 400 and 4000 cm^–1^.

### 4.5 Antifogging

Antifogging tests were performed by holding a sample of PC or PMMA over hot water for 10 s. Then, it was removed and photographed immediately.

### 4.6 Detergent-Free Cleaning

Detergent-free cleaning of plastics was tested by dispensing hexadecane droplets onto the samples, followed by rinsing with DI water. Hexadecane was dyed by adding oil red to enhance the visual contrast.

## Supporting Information Available

Chemical structure of PFPE Zdol; contact angles of Zdol-coated plastics with different treatment order and UV irradiation time; FTIR spectra of Zdol-coated PMMA, PS, and PC with and without UV/Ozone treatment; film thickness of Zdol on plastics; AFM images of plastics with different treatments, transmission intensity of functionalized plastics after antifogging tests; contact angles of functionalized plastics before and after mechanical wiping and water immersion tests (PDF)

## Abbreviations

PMMA: poly (methyl methacrylate)
PS: polystyrene
PC: polycarbonate
PFPE: perfluoropolyether
WCA: water contact angle
HCA: hexadecane contact angle
