# Supplementary material for: How to Make Plastic Surfaces Simultaneously Hydrophilic/Oleophobic?
Source: ACS Appl Mater Interfaces. 2023 Jun 16;15(25):31092–9. doi: 10.1021/acsami.3c06787 (PMC10316401; doi:10.1021/acsami.3c06787)
Supplement: Supplementary file 1 — am3c06787_si_001.pdf [file am3c06787_si_001.pdf]

Supporting Information

How to Make Plastic Surfaces Simultaneously Hydrophilic/Oleophobic?

*Yihan Song, Michaela Dunleavy and Lei Li**

Department of Chemical & Petroleum Engineering, University of Pittsburgh, Pennsylvania 15261, United States

*Corresponding author. Email: lel55@pitt.edu

Experimental Procedure:

Ellipsometry: The thickness of Zdol coating on PMMA and PC is measured by ellipsometry using a J.A. Woollam alpha SE spectroscopic ellipsometer. Before dip coating, the roughness and optical constants of bare plastics are firstly determined using the Cauchy dispersion model.[1] After dip coating, the thickness of Zdol polymer film is determined by subtracting the measured roughness from the thickness of a second Cauchy layer added, holding the same optical constants.

The transmission intensity of PMMA and PC substrate is determined using the same ellipsometer under Transmission mode at normal incidence through each plastic substrate. A S-T baseline scan is performed at the beginning, and then plastic samples before or after anti-fogging tests are held vertically in front of the detector iris for each data scan. The reported transmission intensity is determined by averaging at least 3 measurements.

Atomic Force Microscopy (AFM): Tapping mode AFM was used to characterize the surface topography of the Zdol polymer film on plastic substrates. The scans were conducted using a Bruker Dimension Icon Atomic Force Microscope at a scan size of 10 μm $\times$ 10 μm or 20 μm $\times$ 20 μm, a scan rate of 1.0 Hz and a resolution of 256. The AFM probe used is a MikroMasch

NSC14/AL BS probe (160 kHz resonance frequency, 5.0 N/m force constant, 8 nm tip radius). All experiments were performed at room temperature.

Mechanical Wiping Test: The mechanical robustness of Zdol-coated plastics with UV/Ozone treatment was tested by a hard wiping method reported previously.[2] Kimwipes Delicate Task Wipers purchased from Fisher Scientific were used. The water and hexadecane contact angles were measured before and after the tests.

Water Immersion Test: The water resistance of Zdol-coated plastics with UV/Ozone treatment was tested by immersing the plastics in DI water for 1 day. The water and hexadecane contact angles were measured before and after the tests.

Zdol (Mn = 4000)

$$HOCH_2CF_2O—[CF_2CF_2O]_m—[CF_2O]_n—CF_2CH_2OH$$

Figure S1. The chemical structure of PFPE Zdol.

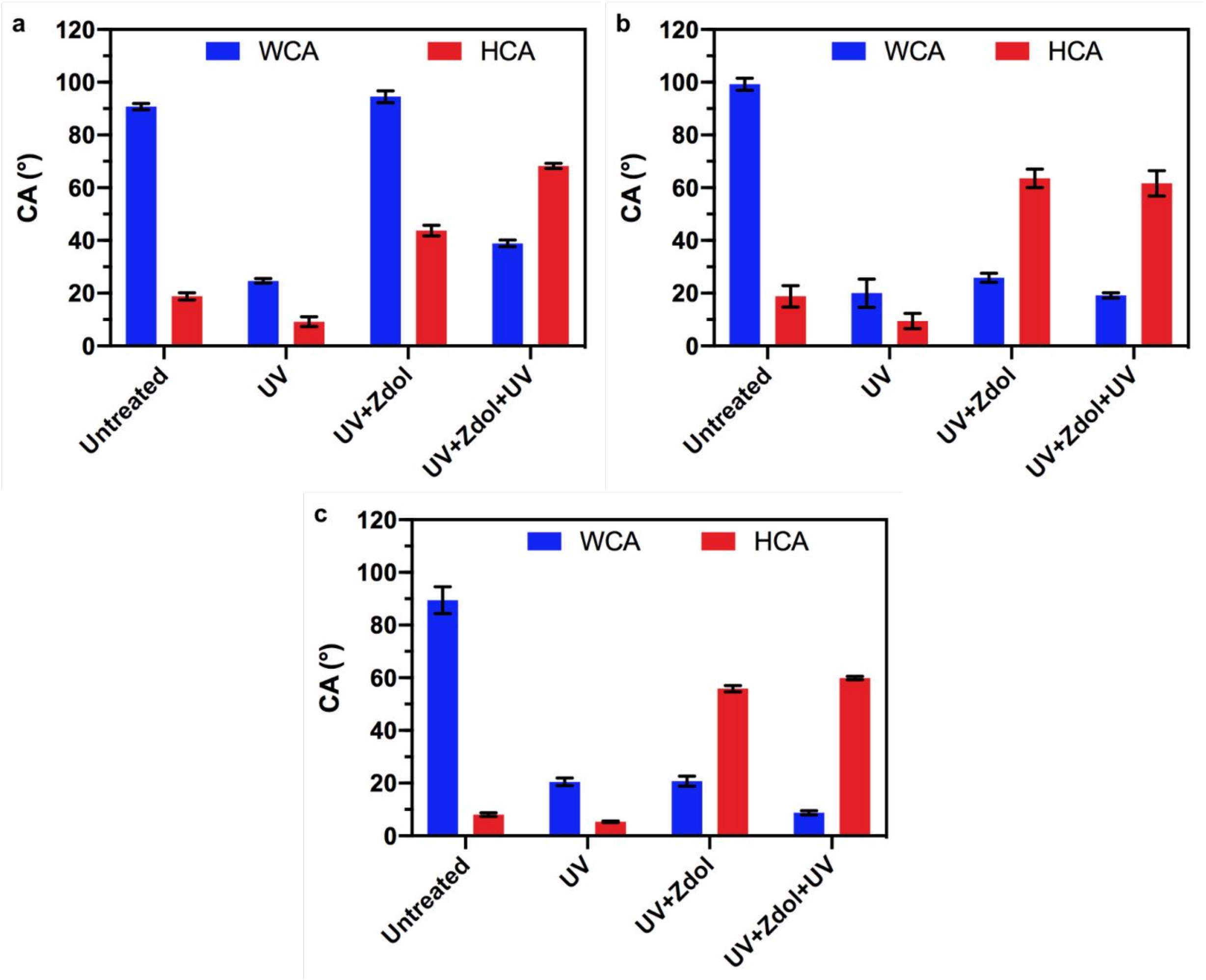

Figure S2. WCA and HCA on PMMA (a), PS (b) and PC (c) with different treatments, including the result of changing the treatment order of Zdol and UV-Ozone.

We have investigated the effect of changing the order of Zdol coating and UV/Ozone treatment. In other words, the plastics are firstly exposed to UV/Ozone for 20 min, followed by dip-coating of Zdol. The contact angle results are shown in Figure S2. Interestingly, PS and PC can be modified with simultaneous hydrophilicity/oleophobicity in this way, whereas PMMA remains hydrophobic/oleophilic with a WCA of > 90° and HCA of ~40°. This could be explained by the fact that the solvent in Zdol solution (Vertrel XF) dissolves the polar groups generated by UV/Ozone on PMMA surface. Thus, to make PMMA simultaneously hydrophilic/oleophobic, a post UV/Ozone treatment is necessary to create polar groups after PMMA is dip-coated by Zdol, as shown in Figure S2a.

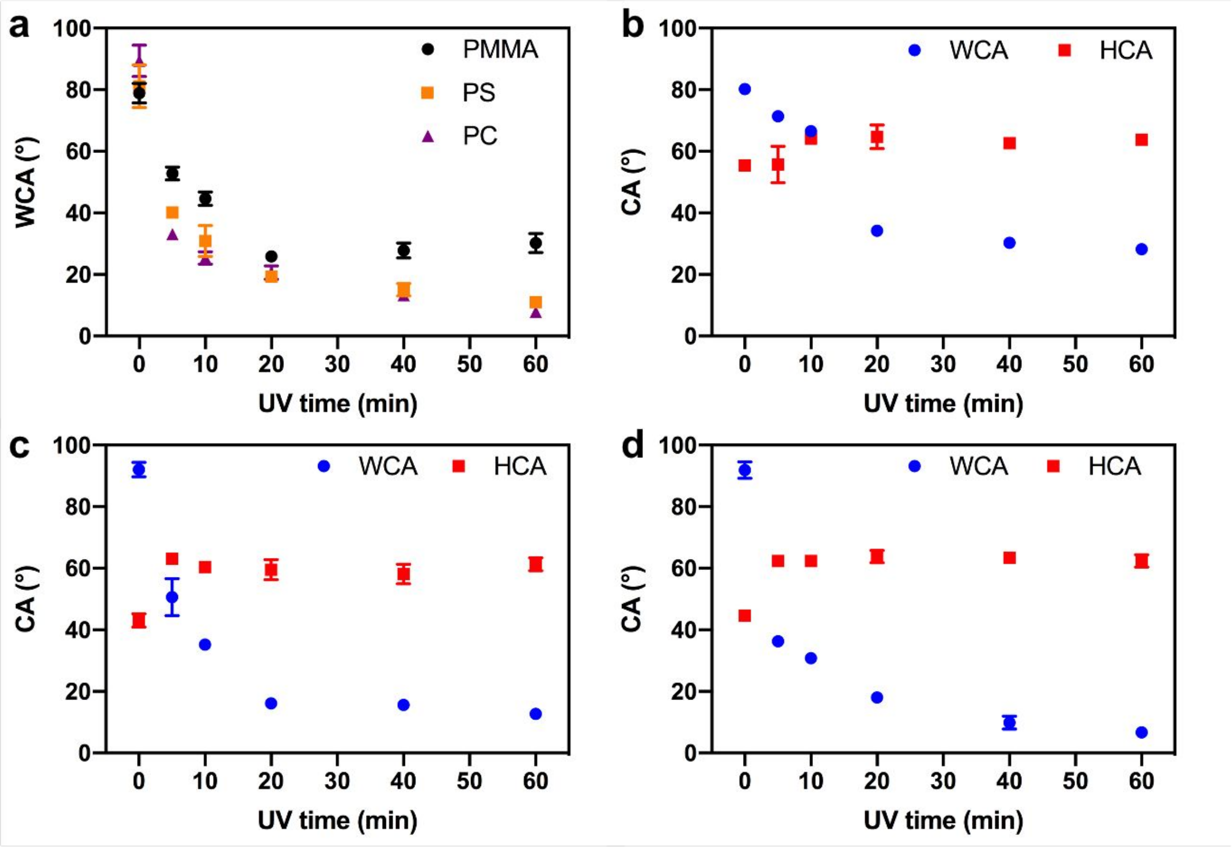

Figure S3. WCA of bare plastics treated with UV-Ozone for different times (a), and the WCA and HCA of Zdol-coated PMMA (b), PS (c) and PC (d) treated with UV-Ozone for different times.

The effect of UV/Ozone treatment time is studied by measuring contact angle of uncoated and coated plastics exposed to different UV/Ozone durations. As shown in Figure S3a, the WCA of PMMA, PS and PC decreases significantly in the first 20 min during UV/Ozone treatment, and longer UV/Ozone treatment gives rise to lower WCA since more and more polar groups are introduced on their surfaces. It takes 20-min UV/Ozone treatment to make PMMA hydrophilic to the point that its WCA is ~30°, which does not decrease further with longer UV/Ozone treatment. On the other hand, shorter UV/Ozone treatment (10 min) is found to be able to decrease the WCA of PS and PC to ~30°, which further drops to ~20° with 20 min treatment and ~10° with 60 min treatment. Similarly, the WCA of Zdol-coated plastics can be decreased by longer UV/Ozone exposure, as shown in Figure S3b-d, and their equilibrium WCA is nearly same as that of uncoated counterpart, indicating that Zdol coating is "neglected" by water and does not affect the

hydrophilicity induced by UV/Ozone treatment. On the other hand, the HCA of Zdol-coated plastics is increased to > 60° after 5 min UV/Ozone treatment, which has induced the bonding between Zdol and plastic substrates so that the configuration of Zdol is more orderly packed. Further increasing UV/Ozone duration does not improve HCA anymore. Therefore, 20-min UV/Ozone treatment is found effective to make all 3 plastics with Zdol coating simultaneously hydrophilic/oleophobic.

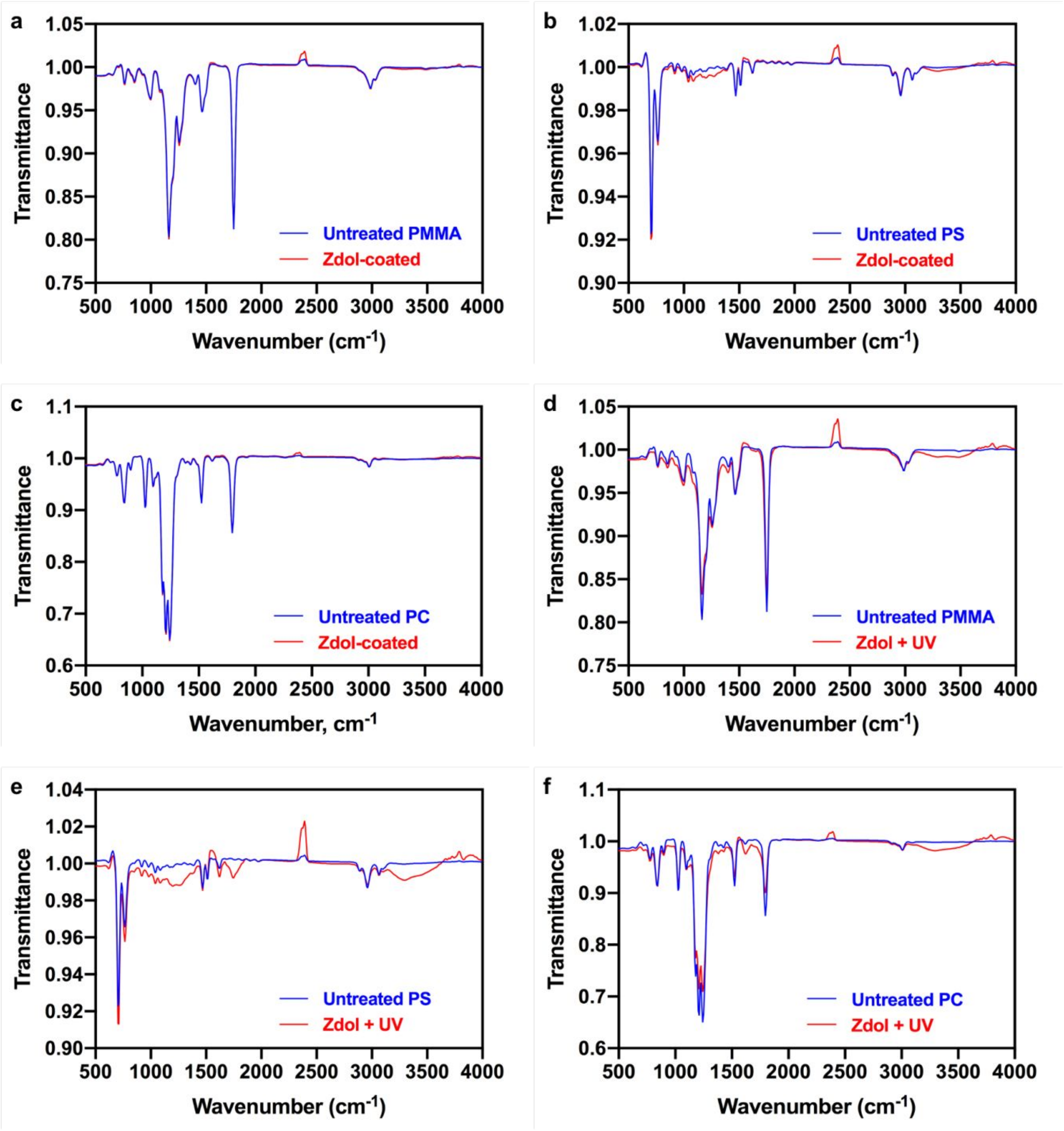

Figure S4. FTIR spectra of Zdol-coated PMMA (a), PS (b) and PC (c) without UV/Ozone treatment, and Zdol-coated PMMA (d), PS (e) and PC (f) with UV/Ozone treatment.

The FTIR spectra of Zdol-coated plastics with and without UV/Ozone treatment can be found in Figure S4. The band intensity in the range of 1000-1300 cm^{-1}, which is assigned to C-F stretching in Zdol molecules,[3] increases in the PS spectrum only, while the uncoated and coated PMMA and PC have almost identical spectra. This is because the infrared absorption of PMMA and PC plastics is much stronger than that of PS so that the sensitivity of a nanometer-thick layer of Zdol on PMMA and PC to infrared irradiation is negligible. Moreover, the regions of C-F stretching and C-O stretching overlap at 1000-1300 cm^{-1}, and the peak growth in PS spectrum due to Zdol coating is smaller than the intensity increase of C-O stretching induced by UV/Ozone treatment. As a result, the spectra of Zdol-coated plastics with UV/Ozone treatment are almost identical to those of uncoated plastics with UV/Ozone treatment, shown in Figure S4d-f and Figure 2. Nevertheless, the effect of Zdol coating followed by UV/Ozone treatment has been apparently verified by the results of contact angle measurements shown in Figure 1.

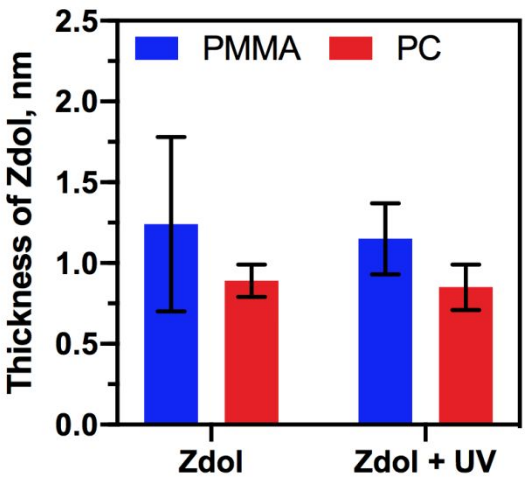

Figure S5. Thickness of Zdol on PMMA and PC with and without UV/Ozone treatment.

The film thickness of Zdol on PMMA and PC was measured by ellipsometry, while the light-scattering surface of PS substrate does not allow such measurement. As shown in Figure S5, the Zdol thickness on PMMA and PC without UV/Ozone treatment is ~1.2 nm and ~0.9 nm

respectively, which is nearly equivalent to the "nanometer-thick Zdol" on silicon wafer reported in our previous studies.[1, 4] After UV/Ozone treatment, the Zdol thickness on both plastic surfaces does not change significantly.

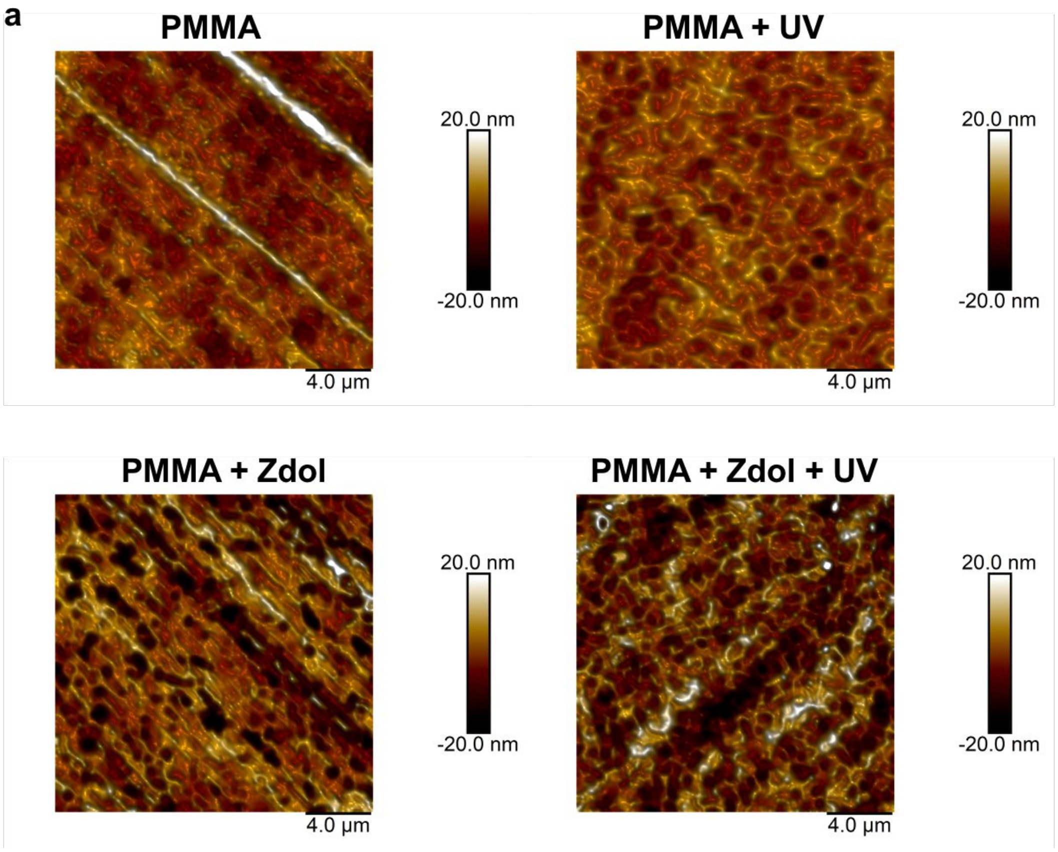

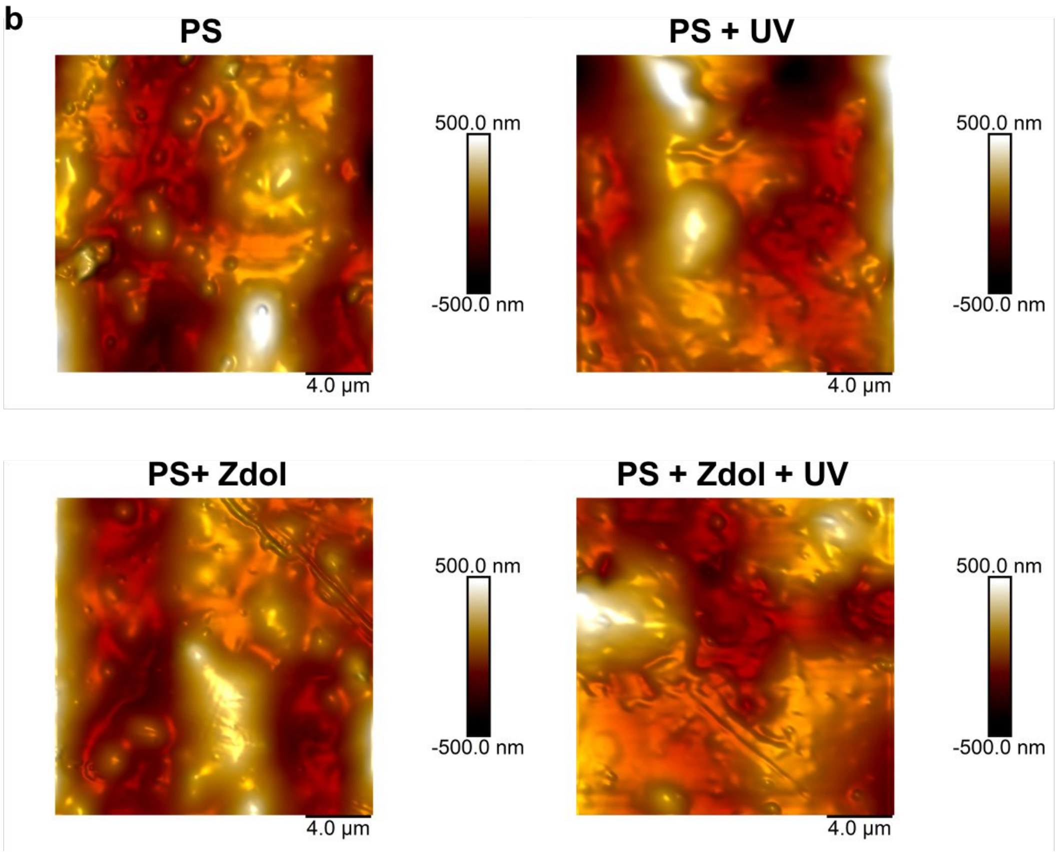
b
PS
PS + UV
PS+ Zdol
PS + Zdol + UV
500.0 nm
-500.0 nm
4.0 μm

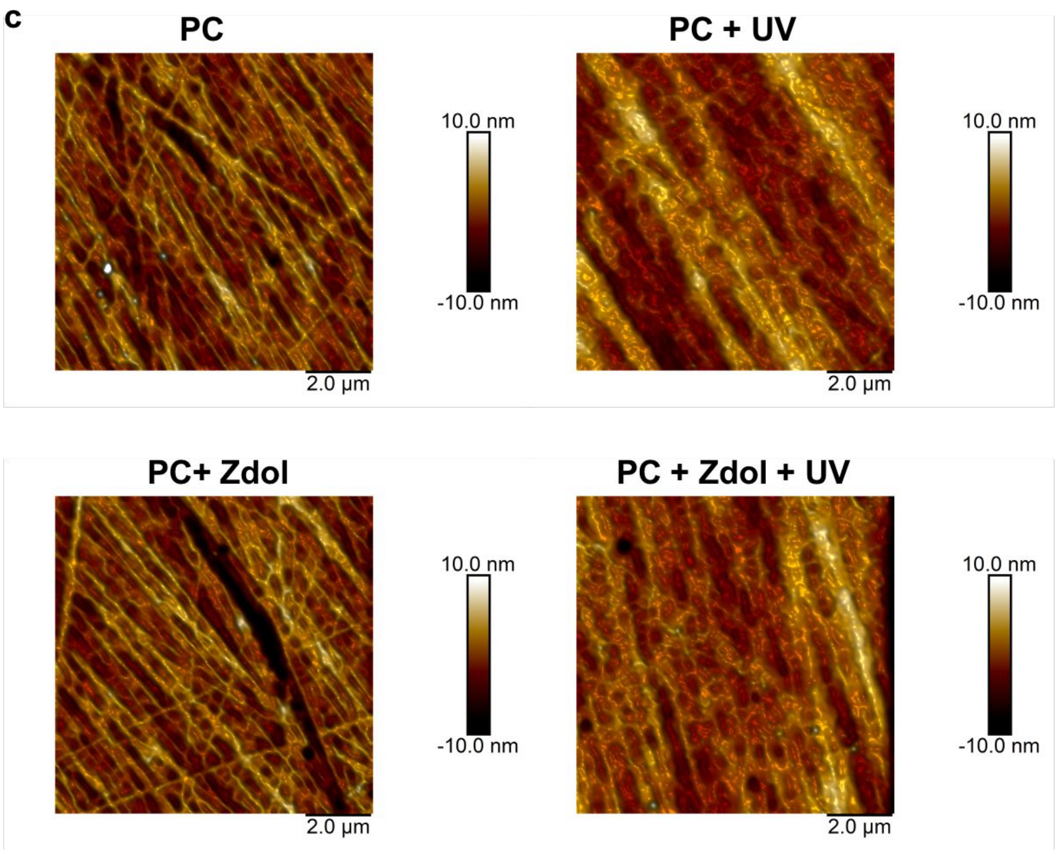
c
PC
PC + UV
PC+ Zdol
PC + Zdol + UV
10.0 nm
-10.0 nm
2.0 μm

Figure S6. AFM images of PMMA (a), PS (b) and PC (c) with different treatments (the scan size is 10 × 10 μm or 20 × 20 μm).

As shown in Figure S6, the AFM topography images show that the surfaces of 3 plastics are rough, and the topographies were not altered significantly after UV/Ozone treatment and Zdol coating. This is because the Zdol polymer film in only nanometer-thick (shown in Figure S5), and its change in molecular level cannot be detected.

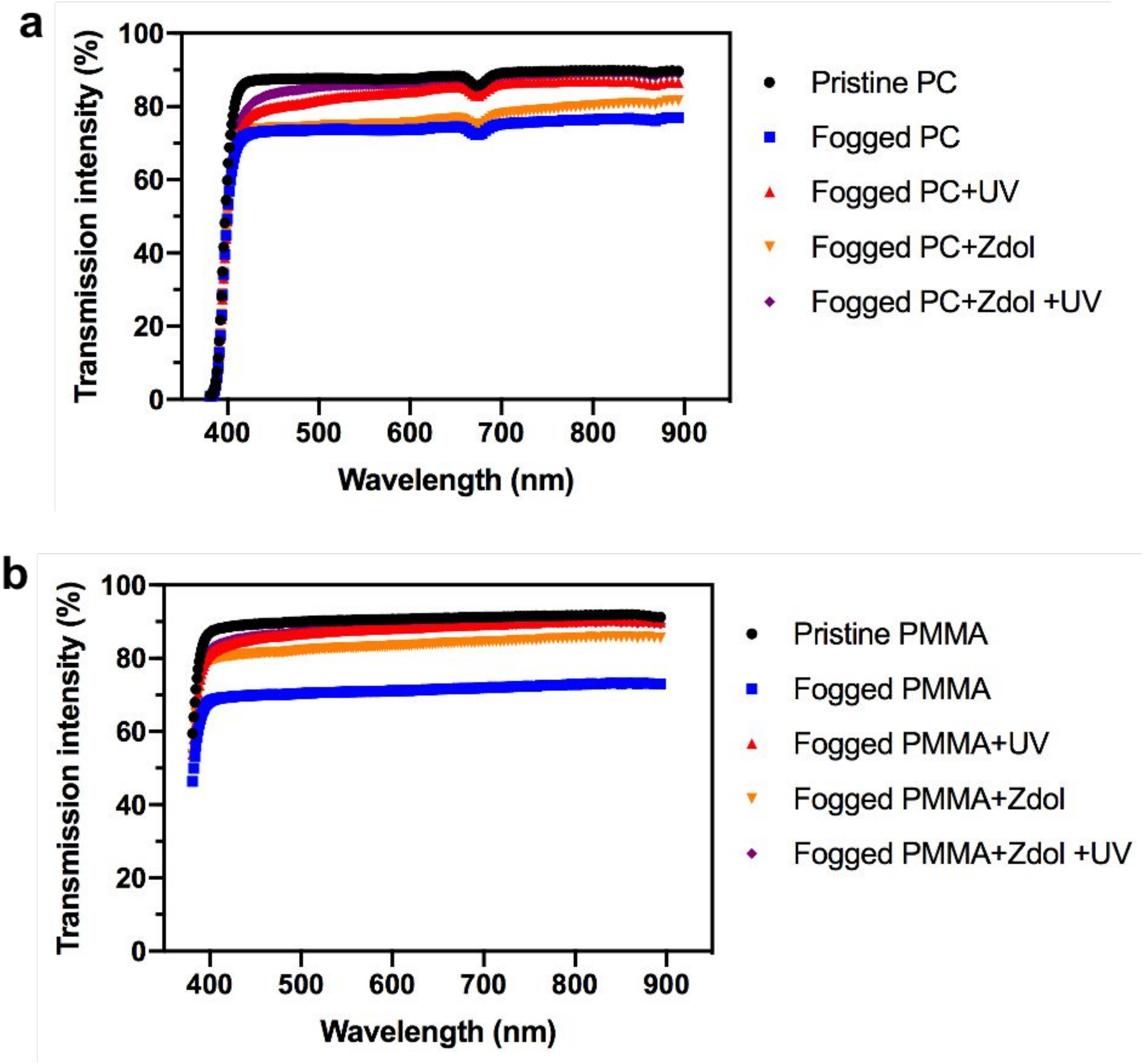

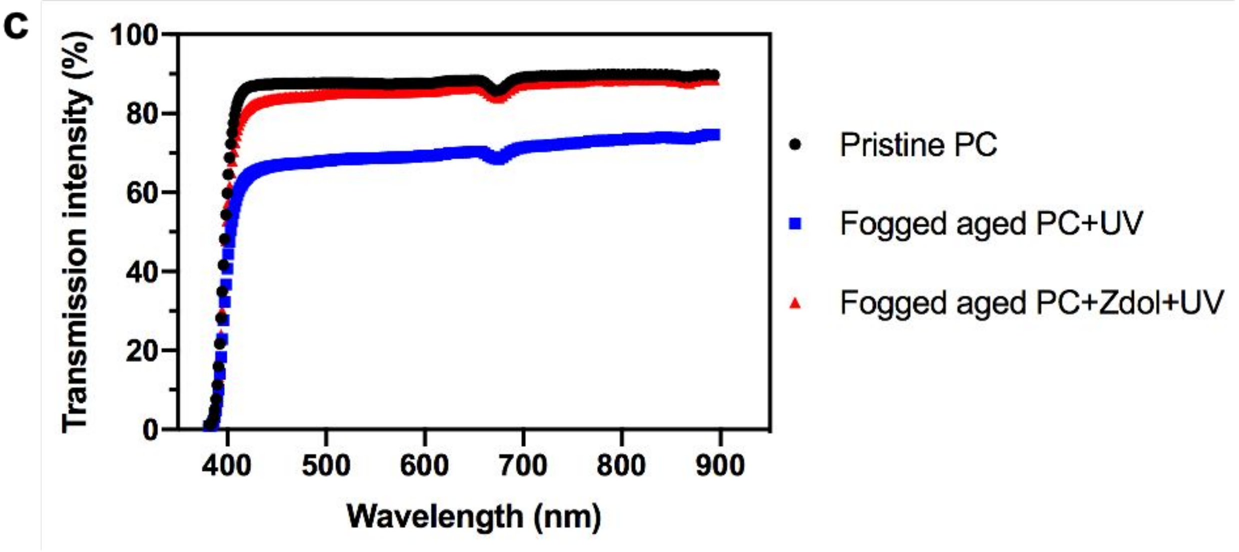

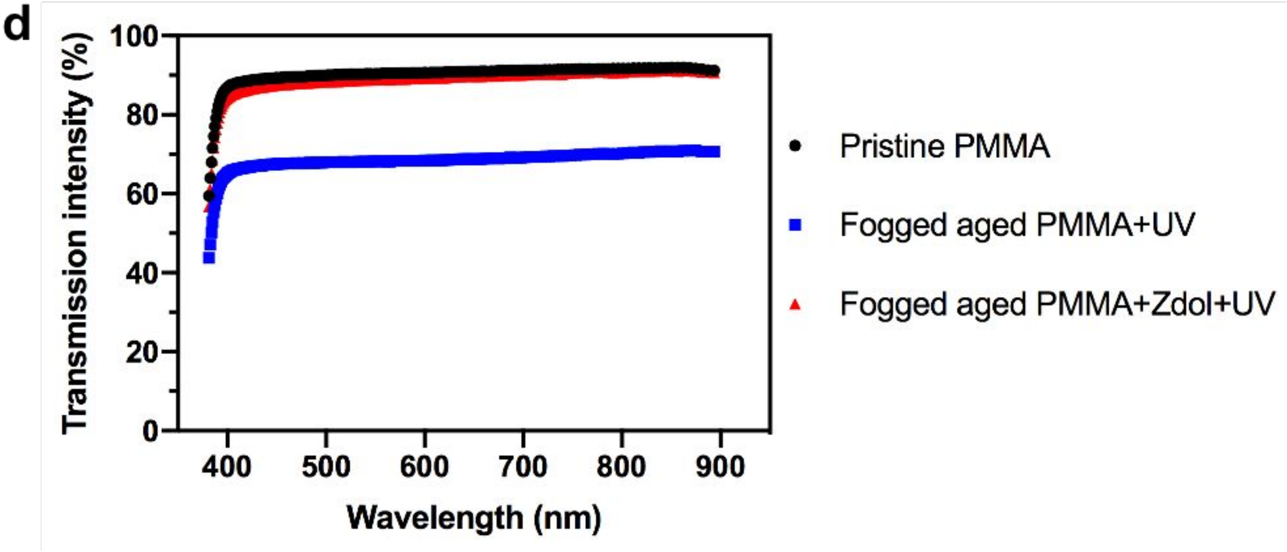

Figure S7. Transmission intensity of freshly functionalized PC (a) and PMMA (b), and aged functionalized PC (c) and PMMA (d), with different treatments before and after anti-fogging tests.

Figure S7a-b reveals the transmission intensity of PMMA and PC samples with different treatments after anti-fogging tests, and the transmission intensity of pristine bare plastic substrates without anti-fogging test is measured as a control. As shown here, the plastics with Zdol coating and UV/Ozone treatment after testing have the transmission intensity (~89% for PMMA and ~88% for PC) similar to that of pristine substrates (~91% for PMMA and ~89% for PC), which indicates the transparency of functionalized plastics is not degraded by fogging. Also, the transmission

intensities of UV-treated PMMA and PC are~89% and ~86%, respectively, which indicates little fogging occurs because of the hydrophilicity of UV-treated plastics. On the other hand, the bare and Zdol-coated plastics show significantly decreased transmission intensity (less than ~82% for all samples), which is consistent with the fact that the hydrophobicity of these plastic cannot inhibit fog formation. In addition, the transmission intensity of aged plastics (uncoated and coated with UV/Ozone treatment) was measured, as shown in Figure S7c-d. The functionalized plastics after aging and anti-fogging tests maintain their transmission intensity (~90% for PMMA and ~88% for PC) similar to that of pristine plastics. Meanwhile the aged UV-treated plastics' (no Zdol coating) transmission intensity decreased to < 75%, which can be attributed to the hydrophobic recovery allowing fogging to occur. Therefore, our functionalized plastics, regardless of being aged or not, exhibit excellent anti-fogging performance.

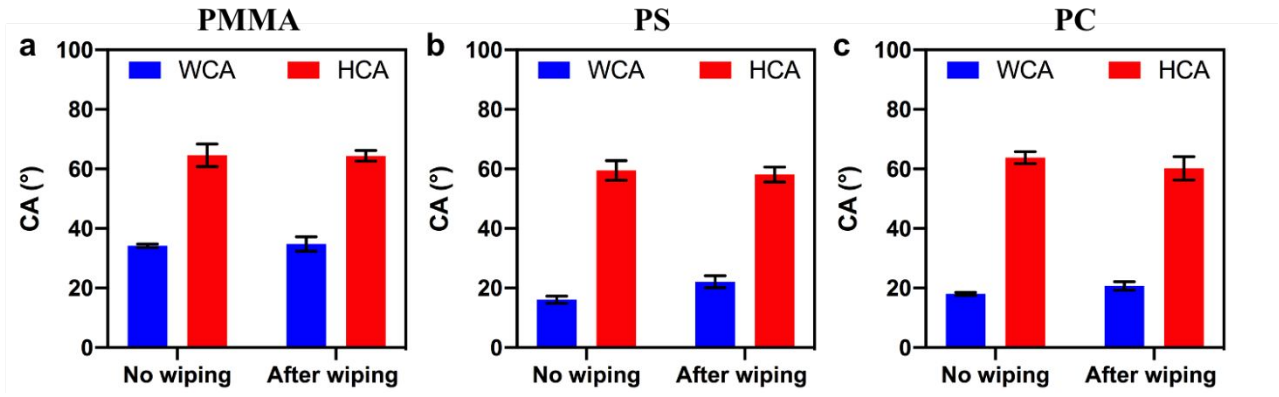

Figure S8. WCA and HCA of functionalized PMMA (a), PS (b) and PC (c) before and after wiping.

The functionalized plastics exhibit excellent mechanical robustness, as shown in Figure S8 where their WCA and HCA remain unchanged after a hard wiping.

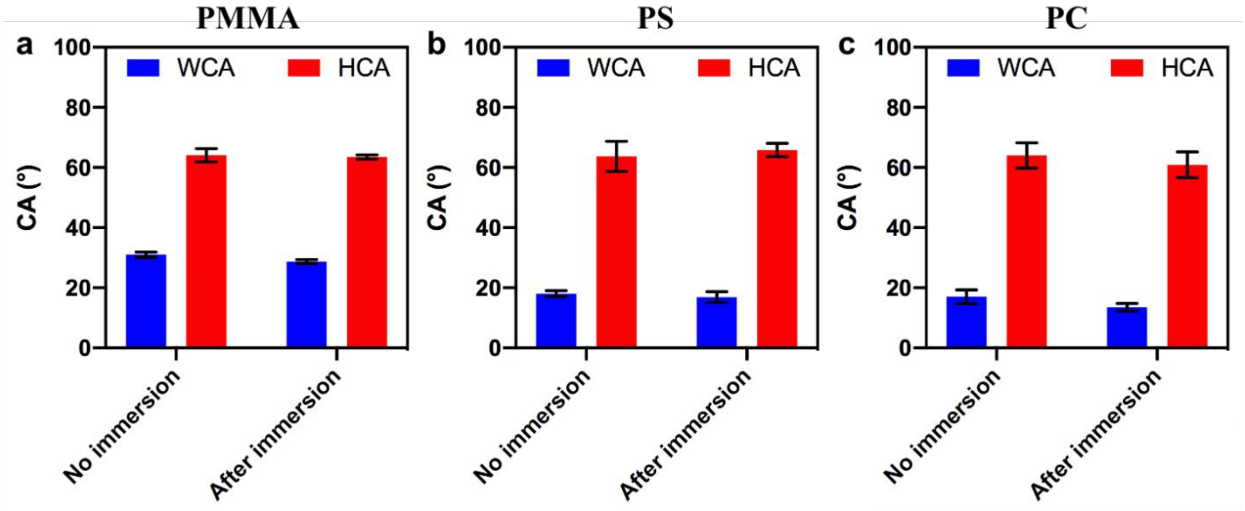

Figure S9. WCA and HCA of functionalized PMMA (a), PS (b) and PC (c) with 2 g/L Zdol before and after immersion in water for 1 day.

The functionalized plastics are immersed in water to test the water resistance. The samples fabricated from 1 g/L Zdol solution have decreased HCA after water immersion for 1 day (data not shown here), indicating the coating can be washed off by water. However, increasing the concentration of Zdol in coating solution can improve the water resistance. As shown in Figure S9, the WCA and HCA of plastics coated with 2 g/L Zdol solution barely change after 1-day water immersion, indicating excellent coating stability in water environment.

References:

1. Wang, Y.; Sun, J.; Li, L., What Is the Role of the Interfacial Interaction in the Slow Relaxation of Nanometer-Thick Polymer Melts on a Solid Surface? *Langmuir* **2012,** *28* (14), 6151-6156.
2. Wang, Y.; Dugan, M.; Urbaniak, B.; Li, L., Fabricating Nanometer-Thick Simultaneously Oleophobic/Hydrophilic Polymer Coatings via a Photochemical Approach. *Langmuir* **2016,** *32* (26), 6723-6729.
3. Li, L.; Jones, P. M.; Hsia, Y.-T., Effect of Chemical Structure and Molecular Weight on High-Temperature Stability of Some Fomblin Z-type Lubricants. *Tribology Letter* **2003,** *16*, 21-27.
4. Li, L.; Wang, Y.; Gallaschun, C.; Risch, T.; Sun, J., Why Can a Nanometer-Thick Polymer Coated Surface Be More Wettable to Water than to Oil? *J. Mater. Chem.* **2012,** *22* (33), 16719-16722.
